# Predictors of cigarette use amongst Pacific youth in New Zealand

**DOI:** 10.1186/1477-7517-10-25

**Published:** 2013-10-17

**Authors:** Tasileta Teevale, Simon Denny, Vili Nosa, Janie Sheridan

**Affiliations:** 1 School of Population Health, University of Auckland, Auckland, New Zealand; 2 Department of Community Paediatrics, School of Population Health, University of Auckland, Auckland, New Zealand; 3 Pacific Health, School of Population Health, University of Auckland, Private bag 92019, Auckland 1142, New Zealand; 4 School of Pharmacy, University of Auckland, Auckland, New Zealand

## Abstract

**Background:**

Despite progressive population health policies to reduce tobacco harm, smoking prevalence continues to be inequitable amongst key ethnic groups in New Zealand. The purpose of this study is to describe the predictors of cigarette use amongst Pacific youth in New Zealand.

**Methods:**

Data were collected as part of Youth’07, a nationally representative survey of the health and well-being of New Zealand adolescents. The study sample comprised 5471 students and this includes 1,178 were Pacific youth.

**Results:**

The smoking prevalence rate for Pacific youth was twice that of New Zealand European youth. Pacific girls and older age groups, ages 16–17, smoked more than Pacific boys and younger adolescents. Pacific youth from higher and mid-deprivation neighbourhoods smoked at twice the rate of youth from low deprivation areas. Local neighbourhood stores (dairies) were the most used location for purchasing cigarettes, and only 12.7% of under-aged adolescents were asked “most of the time” for age identification. Pacific adolescent smoking was associated with parental smoking, peer-group smoking and binge drinking. Parents not knowing the whereabouts of adolescents during after-school hours and night-times were also associated with adolescent smoking. A majority of Pacific adolescent smokers (70.2%) had tried to quit smoking.

**Conclusion:**

The strategies for addressing ethically the issue of *equal health for all* is to allocate increased public health investments towards targeted quit-smoking treatment programmes for Pacific youth in New Zealand. Further qualitative studies with Pacific youth to inform the development of culturally-appropriate youth-focused quit-substance interventions is recommended.

## Background

Tobacco is the second major cause of death in the world and is currently responsible for the death of one in ten adults’ worldwide (about 5 million deaths per year) [1]. World Health Organization (WHO) estimates that, if current smoking patterns continue, it will cause some 10 million deaths every year by 2020, while half the people that smoke today will eventually be killed by tobacco [1].

In the New Zealand context, despite decades of comprehensive tobacco control efforts, tobacco use remains the largest preventable cause of death and disease and in line with other countries, there are large differences in the smoking prevalence between major ethnic groups [1,2]. Although New Zealand has achieved an overall reduction in tobacco use, the gap in smoking prevalence between lower and higher SES groups and between different ethnic groups has increased [3]. Compared to European New Zealanders (smoking prevalence for men 16%; women 17%), Maori (the indigenous people of New Zealand, smoking prevalence for men 38%; women 44%) and Pacific Peoples (diaspora from South Pacific island nations of Samoa, Cook Islands, Tonga, Niue, Tokelau, Fiji and other smaller islands; smoking prevalence for men 28%; women 25%) have substantially higher smoking prevalences [3,4].

Youth ethnicity patterns are comparable to those for adults in New Zealand, although the overall number of young people smoking is considerably smaller than for adults [5,6]. In addition, data from the national youth surveys indicate that these disparities are persisting over time. For example, in 2001 14% of Pacific youth (ages 13–17 years) and 7% of their New Zealand European peers were classified as smokers [7]. By 2007, the prevalence decreased for both groups to 12% and 6% respectively, but the prevalence gap still persists between the two groups [8]. The annual national Year 10 student surveys (students aged 14–15 years), showed that in 1999, 23.0% of Pacific students were smokers compared to 13.1% of NZ European students. In 2008, 10.5% of Pacific students were smokers compared to 4.1% of NZ European students [5]. These disparities contribute to significant health inequalities between ethnic and socio-economic groups and more attention is required to address tobacco use amongst key ethnic groups, who may not be experiencing beneficial effects from population-wide tobacco control interventions [9,10].

This study contributes to the call for more comprehensive data on tobacco use amongst Pacific people, who are a key ethnic group currently experiencing health disadvantages [11]. Tobacco research has so far tended to highlight the predictors of tobacco use amongst Pacific adults [4,12-16]. To the authors’ knowledge, this is the first study examining the predictors of cigarette use amongst Pacific youth in New Zealand.

## Methods

### Survey background

Data for the current study were collected as part of Youth’07, a nationally representative sample of the health and wellbeing of secondary school students in New Zealand. First, 115 schools were randomly selected and 96 agreed to participate in the survey, representing an 84% response rate for schools. The participating schools reflected the general characteristics of secondary schools in New Zealand [17]. From the participating schools, students (n = 12,355) were randomly selected from the school roll and invited to participate. Of these, a total of 9,107 students took part and formed the final Youth’07 sample, representing a 74% response rate and 3.4% of the total 2007 New Zealand secondary school roll.

The survey included a 622 item multimedia questionnaire administered on a Nokia internet tablet. On the day of the survey, students were asked to come to a designated room and upon arrival students were given an anonymous login code to access the questionnaire and identification of their census meshblock number (based on their residential address) to determine the extent of their neighbourhood deprivation. The multimedia nature of the questionnaire meant that all students could read each question and response options themselves, while listening to the questions and responses being read aloud through headphones.

The University of Auckland Human Subject Ethics Committee granted ethical approval for the study. Students and their parents were provided with information sheets about the survey. Students consented themselves to participate in the study on the day of the survey. A more detailed description of the research methodology can be obtained elsewhere [17].

Secondary analysis of the data provided by Pacific students (13% of the total sample) was undertaken. Ethnicity was recorded using New Zealand 2006 Census ethnicity question whereby participants select all of the ethnic groups that they identified with [18]. All students who self-identified any of their ethnic groups as Samoan, Cook Islands, Tongan, Niue, Tokelauan, Fijian, or Other Pacific Peoples were amalgamated to the Pacific grouping, and are included in these analyses (*n* = 1178). New Zealand European students identified through ethnic prioritisation (i.e., students that are non-Maori, non-Pacific, non-Asian) were included in the analyses (*n* = 4797). Intra-Pacific ethnicity analyses could not be completed as findings may be confounded by small sample numbers.

### Outcome measures

To assess smoking prevalence, adolescents were asked the question *“Have you ever smoked a whole cigarette?”* and if they indicated “Yes”, a further branching question was presented; *“How often do you smoke cigarettes now?”* This frequency question had five response options; (1) never – I don’t smoke now; (2) occasionally; (3) once or twice a month; (4) once or twice a week; (5) most days. These five responses were dichotomised into two categories, with adolescents choosing options (2) – (5) classed as current **smokers** and those choosing option (1) as non-smokers.

#### Smoking contexts

To find out where adolescents were obtaining cigarettes, they were asked *“When you smoke cigarettes how do you usually get them?”* Participants were presented with nine choices, with the ability to choose multiple options. Eight of these were used in this study; (1) I buy them myself; (2) I get them from friends; (3) I get them from brothers and/or sisters; (4) I get them from parent(s); (5) I get them from another adult I know; (6) I get someone else to buy them for me; (7) I pinch them; (8) I get them from a tobacco vending machine. The last response option “none of these” was excluded from the analyses.

Adolescents who purchased cigarettes themselves were asked to indicate where they most often bought their cigarettes, choosing from six options: (1) supermarket; (2) dairy; (3) pub; (4) vending machine; (5) petrol station; (6) other people. As the legal age of tobacco purchasing in New Zealand is eighteen years old, a question on showing age identification (ID) when purchasing cigarettes was included; *“When buying cigarettes are you ever asked to show ID?”* Four options were available: (1) almost never; (2) hardly ever; (3) sometimes; (4) most of the time.

A question on age of first smoking occasion was included in the study, as was a question on whether adolescents had ever tried to cut down or give up smoking cigarettes.

#### Home & social environments factors

Adolescents were asked whether their *parent(s) at home* used *cigarettes, tobacco*, and this question was repeated for *friends*. Home environment items used in analyses were about whether parents had knowledge of the adolescents’ *friends, whereabouts after school,* and *whereabouts at night time*.

#### Alcohol binge drinking factors

*Binge drinking* was measured by a series of branching questions. First, adolescents were asked *“Have you ever drunk alcohol (not counting a few sips?)”* If they responded yes, they were asked “*In the past 4 weeks, how many times did you have 5 or more alcoholic drinks in one session within 4 hours?”* with response categories *“*none at all’, “once in the past 4 weeks”, “two or three times in the past 4 weeks”, “every week”, or “several times a week”. Those who reported drinking 5 or more alcohol drinks in one session at least once in the last 4 weeks were classed as binge drinkers (*n* = 305). A question on age of first alcohol drinking occasion was included in the study.

#### Demography

*Age*, *gender* and *ethnicity* were determined by self-report. *Small area deprivation* (NZDep) was determined using the 2006 New Zealand Deprivation Index [19]. For descriptive purposes, the NZDep Index deciles were categorized into three groups reflecting low deprivation (1–3), middle levels of deprivation (4–7), and high deprivation (8–10).

#### Analysis

Frequencies and percentages were used to describe the characteristics of students. Chi-square tests were used to investigate the bivariate associations between ethnicity and outcome variables. Adjusted odds ratio (OR) was estimated using logistic regression models controlling for age, sex and socio-economic deprivation. All analyses were conducted using the procedures in the SAS software v9.2 (Cary, NC) and accounted for the clustered design of the data.

## Results

Table 1 shows that Pacific youth were smoking at twice the prevalence rate (11.9%) of NZ European young people (5.6%) and that this difference is significant (p < 0.001). Pacific adolescent girls were smoking at almost twice the rate (15.4%) of Pacific adolescent boys (8.5%, p = 0.001). Older Pacific youth aged 16–17 years smoked cigarette more than younger adolescents’ aged 13–14 years (p = 0.02). Pacific youth from higher and mid-deprivation neighbourhoods smoked at twice the rate of youth from low deprivation areas, although possibly due to small numbers of Pacific youth from low deprivation neighbourhoods this was not statistically significant (p = 0.29).

**Table 1:** Cigarette use by key demographic variables amongst Pacific and NZ European adolescents

	**Pacific**				**NZ European**			
	**(N = 982)**				**(N = 4489)**			
	***n***	**%**	**95% CI**	**p-value**	***n***	**%**	**95% CI**	**p-value**
Smoking prevalence	117	11.9	9.5 – 14.4		253	5.6	4.9 – 6.3	<.0001
**Gender**								
Males	43	8.6	5.5 – 11.7		114	4.7	3.7 – 5.7	
Females	74	15.5	12.3 – 18.6	0.001	139	6.6	5.5 – 7.7	0.02
**Age**								
13 years	19	7.8	4.0 – 11.7		23	2.6	1.5 – 3.8	
14 years	25	11.9	7.9 – 15.9		44	4.1	2.7 – 5.6	
15 years	20	9.7	6.0 – 13.3		68	6.9	5.1 – 8.6	
16 years	31	16.3	10.5 – 22.0		68	7.8	5.9 – 9.8	
17 years	22	16.7	9.2 – 24.2	0.02	50	7.1	5.3 – 8.8	<.0001
**NZ Deprivation**								
Low	6	6.5	0.8 – 12.3		101	4.9	3.9 – 5.8	
Mid	30	12.2	7.7 – 16.6		107	5.9	4.8 – 6.9	
High	80	12.8	9.5 – 16.1	0.29	43	7.6	5.2 – 10.0	<.0001

### Smokers source of cigarettes

Most Pacific adolescents (77.4%) indicated that they received their cigarettes from *their friends.* The legal age for purchasing cigarettes in New Zealand is eighteen years old, yet almost half (40.8%) of the adolescents in this study said they *purchased cigarettes themselves*. About one in three chose options of other people buying cigarettes for them; *I get someone else to buy them for me* - 33.8%; *I get them from brothers and/or sisters* – 30.5%; *I get them from another adult I know –* 28.7%. One in four adolescents (24.4%) disclosed that they stole cigarettes for use (*I pinch them*) and16.2% of Pacific adolescents indicated that they got cigarettes *from their parent.* Relatively few students, 6.1% used a *tobacco vending machine* to get cigarettes.

### Locations for purchasing cigarettes

Adolescents who purchased cigarettes themselves were asked to indicate where they most often bought their cigarettes, choosing from six options. Table 2 shows that local neighbourhood dairies (stores) were the most frequent locations for adolescents purchasing cigarettes (55.1%). Almost one in four (23.1%) adolescents would buy cigarettes from other people, and 12.6% chose petrol stations, as their most often used place for purchasing tobacco. As tobacco purchase in New Zealand is legally restricted for under-eighteen year olds, adolescents were asked when buying cigarettes, if they were asked identification (ID) to confirm their age. Only one in eight (12.7%) adolescents revealed they were asked for age identification *most of the time* and the majority of students (87.3%) were *sometimes; hardly ever;* and *almost never* asked for ID at the time of purchasing cigarettes.

**Table 2:** Sources and locations of cigarette purchasing amongst Pacific adolescents

**Place adolescents most often buy cigarettes**	***n***	**%**	**95% CI**
Dairy	48	55.1	45.0 – 65.2
Other people	20	23.1	14.0 – 32.1
Petrol Station	11	12.6	6.0 – 19.2
Supermarket	6	6.9	1.3 – 12.4
Pub	2	2.3	0.0 – 5.7
Vending machine	0	0.0	-
**When buying cigarettes are you ever asked to show ID?**	*n*	%	
Almost never	39	44.8	35.0 – 54.5
Hardly ever	17	19.6	10.3 – 28.8
Sometimes	20	22.9	14.1 – 31.7
Most of the time	11	12.7	6.6 – 18.7

Table 3 shows results of the analysis undertaken to test whether Pacific adolescent smoking is associated with parental and peer cigarette use, parental monitoring and alcohol binge drinking behaviours. The analyses revealed that Pacific adolescents were smokers if their parents were smokers and if their friends were smokers. The odds ratio revealed that Pacific adolescents were almost 3 times more likely to smoke cigarettes if they had a parent who was also a smoker (OR = 2.6; 95% CI1.8 – 3.8) and almost all Pacific adolescent smokers indicated that their friends were also smokers, highlighting a strong association amongst social peers and smoking behaviours.

**Table 3:** Prevalence and adjusted odds of Pacific adolescent cigarette smoking by selected home, social and alcohol use variables

	**Cigarette smoking (N = 117)**		**Not cigarette smoking (N = 856)**				
	***n***	**%**	***n***	**%**	Odds ratio*	**95% CI**	p-value*
Parents smoke	77	65.7	361	42.2	2.6	1.8 – 3.8	<0.001
Friends smoke	112	95.7	525	61.4	10.6	4.4 – 26.2	<0.001
Parents have knowledge of where student goes to after school	82	74.5	772	92.3	0.25	0.12 – 0.48	<0.001
Parents have knowledge of where student goes at night time	71	65.7	681	85.1	0.31	0.18 – 0.56	<0.001
Parents have knowledge of who students’ friends are	91	80.5	742	87.1	0.5	0.28 – 0.89	0.02
Binge-drink	78	71.1	219	26.5	6.5	3.8 – 11.0	<0.001

*adjusted for age, gender and deprivation.

In terms of parental monitoring, there was a significant association between smoking and low parental monitoring, that is, parents not knowing the whereabouts of their youth during after school hours or at night times and who their adolescents’ friends were, had an increased likelihood for adolescent smoking.

There was a significant association between alcohol binge drinking and smoking, with almost three-quarters of smokers (71.1%) revealing that they also engaged in binge drinking alcohol. Adolescents were asked to identify *how old they were when they first smoked a whole cigarette*, and *how old they were when they first drank alcohol*. The mean age was calculated to assess which substance use behaviour came first. The mean age for the first smoking occasion was 12.1 years (standard error of mean 0.16, 95% CI for mean 11.79 – 12.41). The mean age for the first alcohol drinking occasion was 12.9 years (standard error of mean 0.11, 95% CI for mean 12.70 – 13.16).

All adolescents who had tried cigarettes once were asked if they had *ever tried to cut down or give up smoking cigarettes?* The majority of students (70.2%) indicated that they had tried to decrease or quit smoking.

## Discussion

This study provides some contexts around cigarette use amongst Pacific youth in New Zealand. We have found that Pacific youth smoke at twice the rate of their New Zealand European counterparts, and higher rates of smoking amongst female Pacific youth and older Pacific youth.

Pacific youth patterns of smoking are unique in gender use, with Pacific girls being much more likely to be smokers than are boys, yet current statistics for adult smokers, indicate that Pacific men use tobacco more than Pacific women [19]. It is not well understood what may be contributing to the differences in gender-related use. Some data suggest that New Zealand born Pacific girls and women are much more likely to be smokers than are Pacific migrant girls and women [13,14,20]. If this is true, it is likely that future prevalences for Pacific women would increase to match men, as the Pacific populations have become increasingly New Zealand born.

The results of the study also reveal that illegal, under-aged cigarette purchasing appears to occur with regularity in amongst Pacific young people in New Zealand. Dairies (small local neighbourhood stores) were the most frequented location for purchasing cigarettes illegally and most youth cigarette purchasing did not require age identification to comply with current laws. This highlights a significant flouting of the law by retailers and a lack of law enforcement. This study supports the strategies to limiting youth access by increasing penalties for vendors who sell under-age cigarettes and to increase the individual purchase price for cigarettes [21]. Increasing the cost of cigarettes has been found to be effective for Pacific young people’s attempts to stop smoking [16].

This study confirmed the salience of parental and peer-use on adolescent smoking behaviours, which affirms previous research on social influences [22-26]. Parental monitoring, that is, having parents who knew the whereabouts of their young people during after-school hours and at night time, decreased the likelihood of youth smoking behaviours. This finding confirms previous research on the role of parental monitoring in decreasing adolescent smoking risk [27-30]. Having a friend who was a smoker was much more likely to influence Pacific youth smoking behaviours and friends who were also smokers were significant suppliers of cigarettes. The significant role of peers with respect to Pacific youth smoking is common amongst adolescents in general and this is likely to be a combination of peer pressure, and ‘peer selection’, where young people choose to be with similar others [30-32].

In addition, alcohol use was also a regular feature for Pacific youth smokers. The co-occurrence of both cigarette and alcohol use is emerging from recent literature [33-35] and its existence for Pacific groups is supported by some data on adults [36]. This study revealed that both substance initiations occurred in the pre-teen years for Pacific young people. The co-occurrence of the use of both substances highlights the need for both prevention and management strategies to be targeted at younger age-groups, and for youth quit-smoke or quit-drink programmes to be offered in tandem with one another. Our study found most Pacific youth smokers had tried to quit smoking, but emerging data highlight a lack of accessibility to quit-substance-use programmes [37]. Pacific participants in a qualitative study indicated a lack of awareness and use of mainstream quit-smoking services and products and that most Pacific people stopped unaided, that is, going “cold turkey” [16]. Youth in particular, had very limited health service knowledge and may be unsupported in their attempts to quit an addictive substance [16].

The strengths of this study include the use of nationally representative data and ability to present Pacific youth health data exclusively. In most cases, ethnic research data is presented in amongst national data sets and publications which make it difficult for researchers to access ethnic-specific information. As health patterns across most OECD countries follow distinct trends, particularly entrenched health disparities across ethnic groups [38-41], it becomes more important to publish health information for each disadvantaged group that may require quite specific and unique resolutions.

A limitation of this study is that it could not complete intra-Pacific analyses due to small sample numbers. Previous research does indicate that different Pacific island groups, like the eastern Polynesian groups of Cook-Islanders and Niueans have much higher smoking rates compared to the western Polynesian groups of Samoans and Tongans [5,42]. However, taken individually, each Island group has substantially higher smoking rates compared to their New Zealand European counterparts and further breakdown by intra-Pacific groups would not add further value to conclusions for addressing youth smoking behaviours proposed here.

## Conclusion

The persistence of unequal rates of cigarette use over time between ethnic groups is concerning and one that calls for both public-health policy approaches and for unique Pacific community-focused approaches. A strategy for addressing ethically the issue of *equal health for all* is to allocate increased public health investments in targeted quit-smoking treatment programmes for Pacific youth in New Zealand. Further qualitative studies with Pacific youth to inform the development of culturally-appropriate youth-focused quit-substance interventions is recommended.

## Competing interests

The authors declare that they have no competing interests.

## Authors’ contributions

All authors were involved in the study design. TT led the writing of the manuscript which was reviewed by all authors who approved the final version of the manuscript. SD led the statistical analysis for the study. All authors read and approved the final manuscript.
